# Increase in the intensity of an optical signal with fluorescein during proton and carbon‐ion irradiation

**DOI:** 10.1002/acm2.13309

**Published:** 2021-06-14

**Authors:** Seiichi Yamamoto, Takuya Yabe, Takashi Akagi

**Affiliations:** ^1^ Department of Integrated Health Science Nagoya University Graduate School of Medicine Nagoya Japan; ^2^ Department of Medical Technology Nagoya University Hospital Nagoya Japan; ^3^ Hyogo Ion Beam Medical Center Tatsuno Japan

**Keywords:** carbon ion, Cherenkov light, fluorescein, imaging, luminescence, proton

## Abstract

**Purpose:**

Although the imaging of luminescence emitted in water during irradiation of protons and carbon ions is a useful method for range and dose estimations, the intensity of the images is relatively low due to the low photon production of the luminescence phenomenon. Therefore, a relatively long time is required for the imaging. Since a fluorescent dye, fluorescein, may increase the intensity of the optical signal, we measured the luminescence images of water with different concentrations of fluorescein during irradiation of protons and carbon ions and compared the results with those by measurements with water.

**Methods:**

A cooled charge‐coupled device (CCD) camera was used for imaging a water phantom with different concentrations of fluorescein from 0.0063 to 0.025 mg/cm^3^, in addition to a water phantom without fluorescein during irradiation of 150‐MeV protons and 241.5‐MeV/n carbon ions.

**Results:**

For both protons and carbon ions, the intensity of the luminescence images increased as the concentration of fluorescein increased. With a fluorescein concentration of 0.025 mg/cm^3^, the intensities increased to more than 10 times those of water for both protons and carbon ions. Although the shape of the depth profiles of luminescence images of water with fluorescein appeared similar to that of water for protons, those for carbon ions were different from those of water due to the increase in the Cherenkov light component at shallow depths by the decrease in the angular dependencies of the Cherenkov light.

**Conclusion:**

We confirmed the increase in intensity of the luminescence of water by adding fluorescein for particle ions. With a small amount of Cherenkov light contamination in the images, such as protons, the relative distributions of the luminescence images with fluorescein were similar to that of water and will be used for range or dose determination in a short time.

## 1 INTRODUCTION

The luminescence imaging of water during irradiation of protons and carbon ions are useful method for range estimations.^1^, ^2^ The luminescence imaging of water are also promising for dose estimation by correcting the contamination of the Cherenkov‐light component in the images.^3^, ^4^ However, the intensities of the luminescence images are relatively low due to the low photon production of the luminescence phenomenon. The produced light per particle of the luminescence is small^2^, ^5^, ^6^, ^7^ compared with Cherenkov‐light^8^, ^9^ or a plastic scintillator.^10^, ^11^ With the low photon production of the luminescence phenomenon, a relatively long time was required for the imaging of the luminescence of water. For the spot‐scanning pencil beam protons, more than 1 min was required to measure a luminescence image of water with a reasonable image quality.^1^ If the intensity of the luminescence images of water were increased, the applications of such imaging would be wider. For example, the imaging of mini‐beams is possible using luminescence imaging, but we used an acrylic block due to the higher intensity needed for this purpose, since the tungsten slits used for the experiments absorbed most of the beams.^12^

Using a fluorescent dye soluble in water is a promising method to increase the luminescence during irradiation to water. A fluorescent dye, fluorescein, was used in an attempt by Glaser et al. to reduce the angular dependencies of Cherenkov light during irradiation of high‐energy X‐rays to water from a medical linear accelerator (LINAC).^13^ In that paper, an increase in the Cherenkov light was also reported with increasing fluorescein concentration.^13^ They also used other fluorescent dye for their optical imaging of Cherenkov light during irradiation of high‐energy X‐rays from a LINAC.^13^, ^14^, ^15^

Since fluorescein may increase the intensity of the luminescence of water, we measured the luminescence images of water at different concentrations of fluorescein during irradiation of protons and carbon ions and compared the results with those measured with water. We also evaluated the depth and lateral profiles of luminescence images of water at different concentrations of fluorescein and compared the results with those of water without fluorescein to evaluate the possibility of using them for range or dose determination.

## 2 MATERIALS AND METHODS

### 2.A Luminescence imaging of water at different concentrations of fluorescein during irradiation of protons or carbon ions

A cooled charge‐coupled device (CCD) camera was used for imaging of the luminescence of water during irradiation of 150 MeV protons. The energy of protons was slightly higher than Cherenkov light threshold for the secondary electrons to emit Cherenkov light (120 MeV). We also used 241.5 MeV/n carbon‐ions. The energy of carbon‐ions was higher than Cherenkov light threshold for the secondary electrons to emit Cherenkov light (120 MeV/n). We measured the luminescence images during irradiation of protons or carbon‐ions to a water phantom with different concentrations of fluorescein from 0.0063 to 0.025 mg/cm^3^, in addition to a water phantom without fluorescein.

**Fig. 1:**
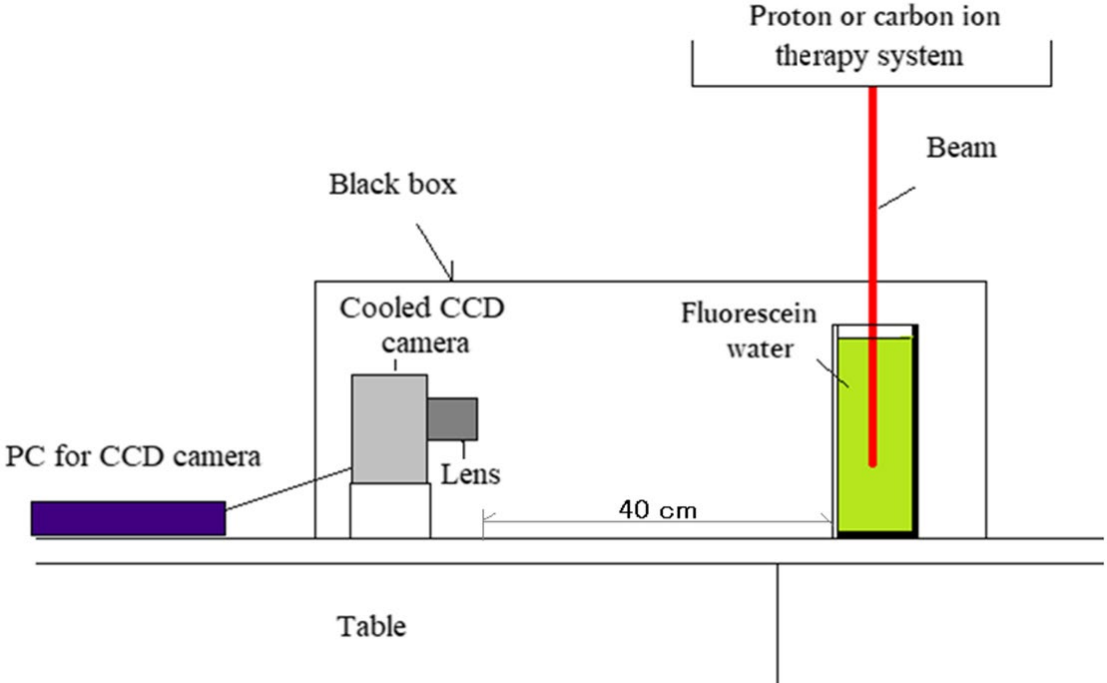
Experimental setup for luminescence images of water at different concentrations of fluorescein during irradiation of protons or carbon ions.

A block diagram of our experiment on the luminescence imaging of water at different concentrations of fluorescein during irradiation of protons or carbon ions is shown in Fig. 1. A phantom containing a solution of water with fluorescein was set on the couch of a proton or carbon‐ion therapy system (Mitsubishi Corp., Japan). This therapy system can irradiate protons as well as carbon ions. A cooled CCD camera (BITRAN BU‐50LN, Japan) equipped with a C‐mount F‐1.4 lens was set 40 cm from the phantom surface. The acrylic water phantom's outer dimensions were 20 cm × 20 cm × 10 cm as shown in Fig. 1.

During exposure of the CCD camera for 2 min, 150‐MeV energy protons or 241.5‐MeV/n carbon ions were irradiated to the water phantom for 5 s for protons (~2 × 10^11^ particles) and 10 s for carbon‐ions (~1.3 × 10^10^ particles). The beam width was ~47 mm full width at half maximum (FWHM) for protons and ~18 mm FWHM for carbon ions. These widths were measured by scanning an ion chamber.

Imaging with the CCD camera was conducted for protons with 2 × 2 binning and the matrix size of the images at 386 × 290. The size of a pixel in the images was 1.0 mm × 1.0 mm. The imaging of the CCD camera for carbon ions was conducted with 1 × 1 binning and the matrix size of the images at 772 × 580. The size of a pixel in the images was 0.5 mm × 0.5 mm. We used images with a larger matrix size for carbon ions because the Bragg peak was expected to be sharper than for protons.

Luminescence imaging of water was made using three different concentrations of fluorescein (F0095, Tokyo Chemical Industry Co., Tokyo, Japan): 0.0063, 0.0125, and 0.025 mg/cm^3^. Fluorescein water of 0.0032 mg/cm^3^ was additionally measured for protons, while fluorescein water of 0.05 mg/cm^3^ was measured for carbon ions. The upper concentration was limited by the precipitation of fluorescein in water. With a fluorescein concertation higher than 0.025 mg/cm^3^, significant precipitation of fluorescein was observed in the bottom of the water phantom. We also measured the luminescence image of water without fluorescein.

### 2.B Monte Carlo simulation of dose distributions of protons and carbon ions

For a comparison with the experimental data measured by optical imaging, dose distribution in the water phantom for protons at 150‐MeV energy or carbon ions at 241.5 MeV/n was simulated using a Monte Carlo code (Geant4 version 10.3.p03).^16^

The dimensions of the water phantoms used as targets were 20 cm × 20 cm × 10 cm. The deposited energy was scored as a two‐dimensional dose distribution with a pixel size of 0.5 mm × 0.5 mm. The applied physics list was set to QGSP_BERT for hadronic processes and G4EmStandardPhysics_opt3 for electron‐magnetic processes. The cut‐range for secondary particles was set to 0.1 mm.

The incident energy of protons or carbon ions was set to 150 MeV or 241.5 MeV/u, and the energy spread was set to 0.1% of the standard deviation of the incident energy. The beam width was 47 mm FWHM for protons and 18 mm FWHM for carbon ions. The number of primary particles was set to 1.0 × 10^6^ for protons and 1.0 × 10^5^ for carbon ions. The distance between the beam source and the phantom surface was set to 2 cm.

### 2.C Light spectra measurement of fluorescein water by irradiation of UV light

We measured the light emission spectra of the fluorescein water by the irradiation of UV light. UV light was used because the experiments were much easier and the spectrum resolution was much higher than the light imaging conducted during irradiation of protons or carbon ions. We irradiated UV light, using a 365‐nm light emitting diode (LED)‐based UV black light (KONTEC, UV‐SVGNC365‐01, Osaka, Japan, wavelength: 365 ± 3 nm, width 7 nm), to the fluorescein water, and the spectra were measured from the side with a spectrometer (Hamamatsu Photonics, C10082CA). Fluorescein water at a concentration of 0.01 mg/cm^3^ was used for the measurement. We also measured the light spectrum for water.

### 2.D Image processing

A public domain software application (ImageJ) was used for the image processing. We eliminated noise spots using Remove Outlier, a function of ImageJ. Then, by subtracting the blank image, the non‐uniformity and offset value were corrected.^1^, ^2^ We measured the depth and lateral profiles from the image having a ~10‐mm width with this software. The average pixel values of the widths were calculated along a line in the luminescence images.

## 3 RESULTS

### 3.A Luminescence images of water at different concentrations of fluorescein during irradiation of protons

**Fig. 2:**
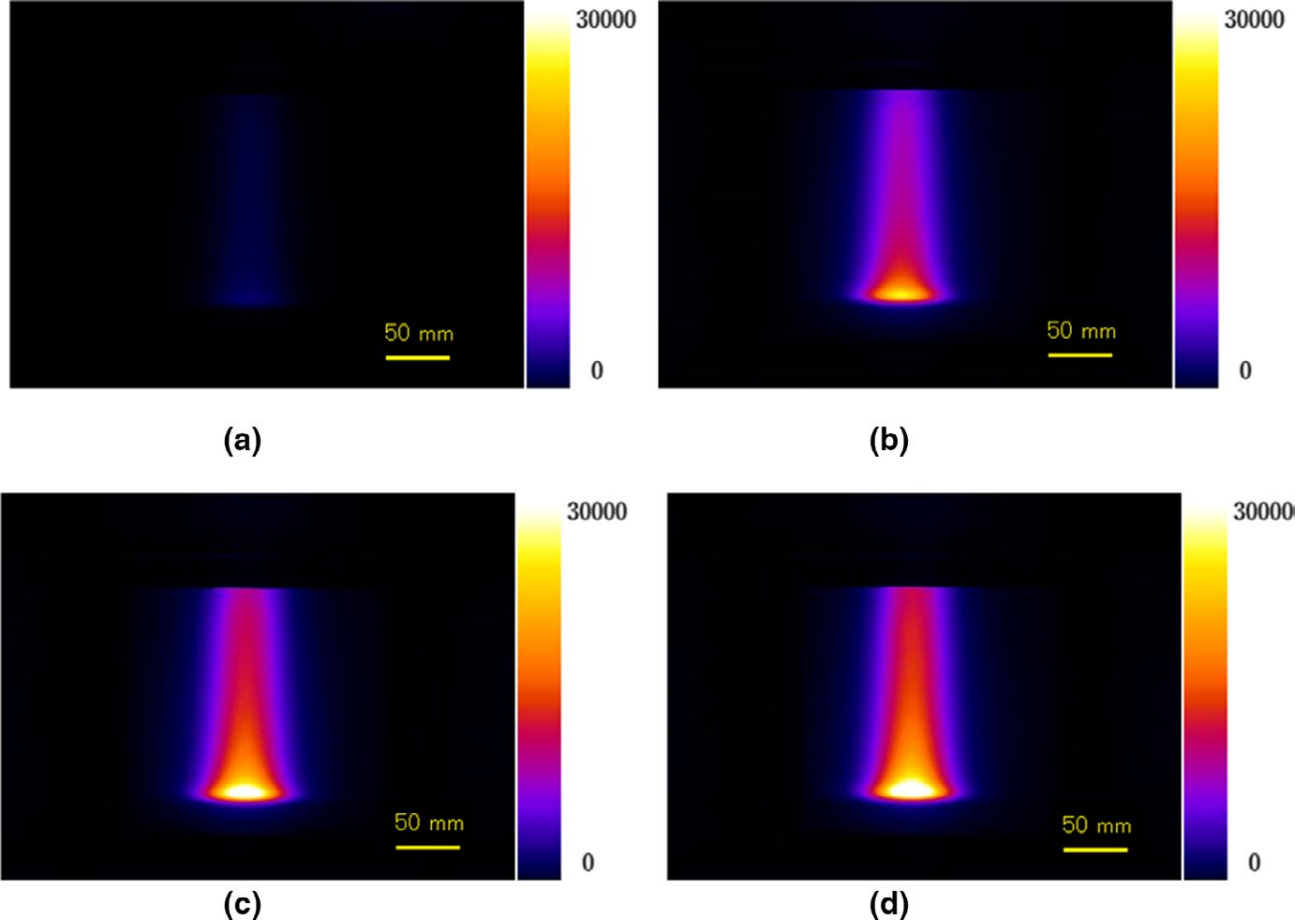
Measured luminescence images during 150‐MeV proton irradiation of water without fluorescein (a) and with fluorescein concentrations of 0.0063 mg/cm^3^ (b), 0.0125 mg/cm^3^ (c), and 0.025 mg/cm^3^ (d).

Figures. 2(a)–2(d) show experimentally measured luminescence images of water during irradiation of protons without fluorescein (a) and with fluorescein concentrations of 0.0063 (b), 0.0125 (c), and 0.025 mg/cm^3^ (d). The luminescence intensity was observed to increase as the fluorescein concentration increased.

**Fig. 3:**
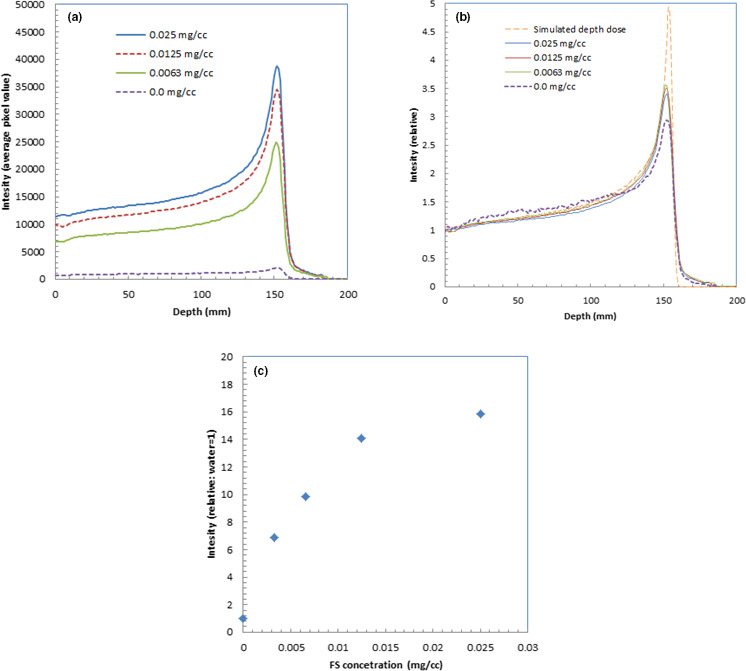
Depth profiles of the measured luminescence images of water during irradiation of 150‐MeV protons with fluorescein at different concentrations (a), relative depth profiles of the measured luminescence images of water with fluorescein at different concentrations (b), and relative intensity of the depth profiles as a function of fluorescein concentration (c).

We show the depth profiles of the measured luminescence images of water with fluorescein at different concentrations in Fig. 3(a). We could observe the luminescence intensity increase as the fluorescein concentration increased.

Figure 3(b) shows the relative depth profiles of the measured luminescence images of water normalized at the shallow part of the depths. These relative depth profiles were normalized at the entrance of the phantoms. The depth profiles were almost the same shape except for water without fluorescein (concentration 0.0 mg/cm^3^). The depth profile for water showed a slightly lower Bragg peak and slightly higher intensity around the mid‐point of the depth. For comparison, we also show a simulated depth dose profile in Fig. 3(b).

Figure 3(c) shows the relative intensity measured for the luminescence images of water as a function of fluorescein concentration, where the intensity of water is normalized to 1.0. The intensities were evaluated by averaging at depths from 0 to 170 mm. The luminescence intensity was more than 10 times higher than that of water for the images at fluorescein concentrations greater than 0.0125 mg/cm^3^.

Table 1 lists the ranges estimated from the luminescence images of water and fluorescein at different concentrations. Ranges were estimated at 80% of the peak for the profiles. The ranges were matched with measured luminescence images of water, fluorescein at different concentrations, and simulation within an error of 1 mm.

**Table 1:** Ranges estimated from luminescence images of water, fluorescein (FS) at different concentrations, and simulation for protons.

	Water	FS: 0.0063 mg/cm^3^	FS: 0.0125 mg/cm^3^	FS: 0.025 mg/cm^3^	Simulation
Range	155 mm	155 mm	156 mm	156 mm	155 mm

**Fig. 4:**
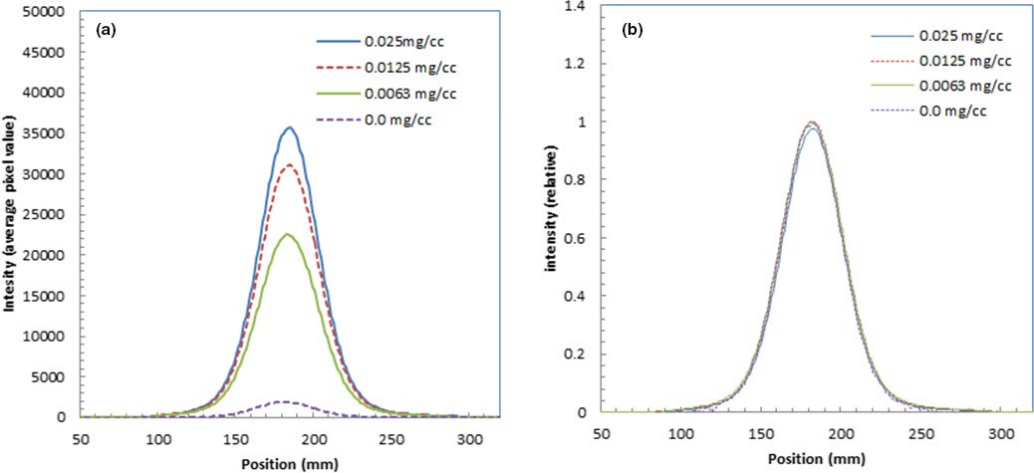
Lateral profiles of measured luminescence images of water during irradiation of 150‐MeV protons at Bragg peak area with fluorescein at different concentrations (a) and relative lateral profiles of the measured luminescence images of water normalized at the peaks (b).

Figure 4(a) shows the lateral profiles of the measured luminescence images of water in the Bragg peak area (152‐mm depth) with fluorescein of different concentrations. We could observe the luminescence intensity increase as the fluorescein concentration increased.

Figure 4(b) shows the relative lateral profiles of the measured luminescence images of water normalized at the peaks. The depth profiles were almost the same shape for all images with fluorescein at different concentrations as well as water.

Table 2 lists the beam widths in FWHM estimated from the luminescence images of water and fluorescein at different concentrations for protons. The differences in the widths were within 1 mm.

**Table 2:** Beam widths estimated from luminescence images of water and fluorescein (FS) at different concentrations for protons.

	Water	FS: 0.0063 mg/cm^3^	FS: 0.0125 mg/cm^3^	FS: 0.025 mg/cm^3^
FWHM	45 mm	46 mm	45 mm	45 mm

### 3.B Luminescence images of water at different concentrations of fluorescein during irradiation of carbon ions

**Fig. 5:**
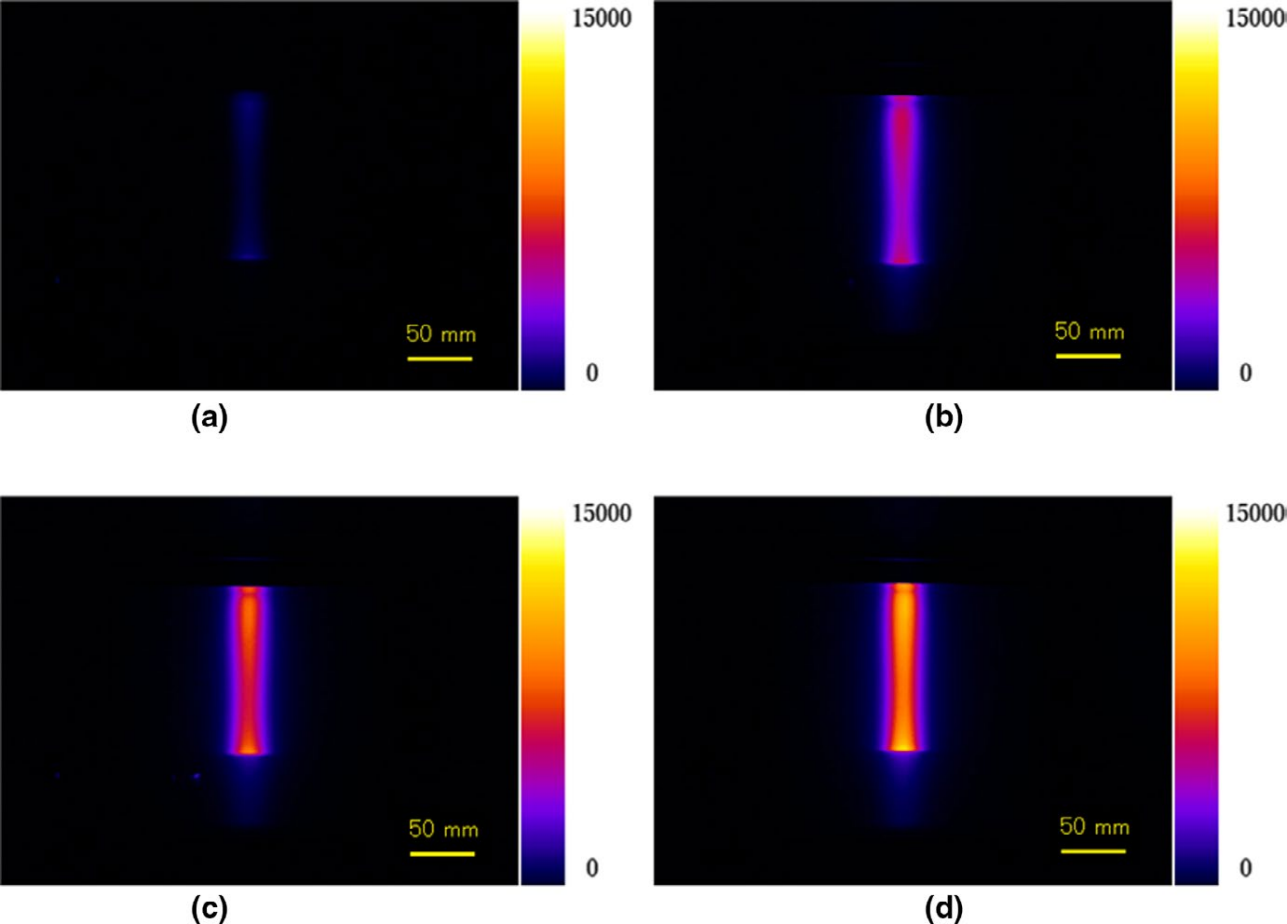
Measured luminescence images during 241.5 MeV/u carbon‐ion irradiation with luminescence images of water without fluorescein (a) and with fluorescein concentrations of 0.0062 (b), 0.0125 (c), and 0.025 mg/cm^3^ (d).

Figures 5(a)–5(d) show experimentally measured luminescence images of water during irradiation of carbon ions without fluorescein (a) and with fluorescein concentrations of 0.0063 (b), 0.0125 (c), and 0.025 mg/cm^3^ (d). We could observe the luminescence intensity increase as the fluorescein concentration increased.

**Fig. 6:**
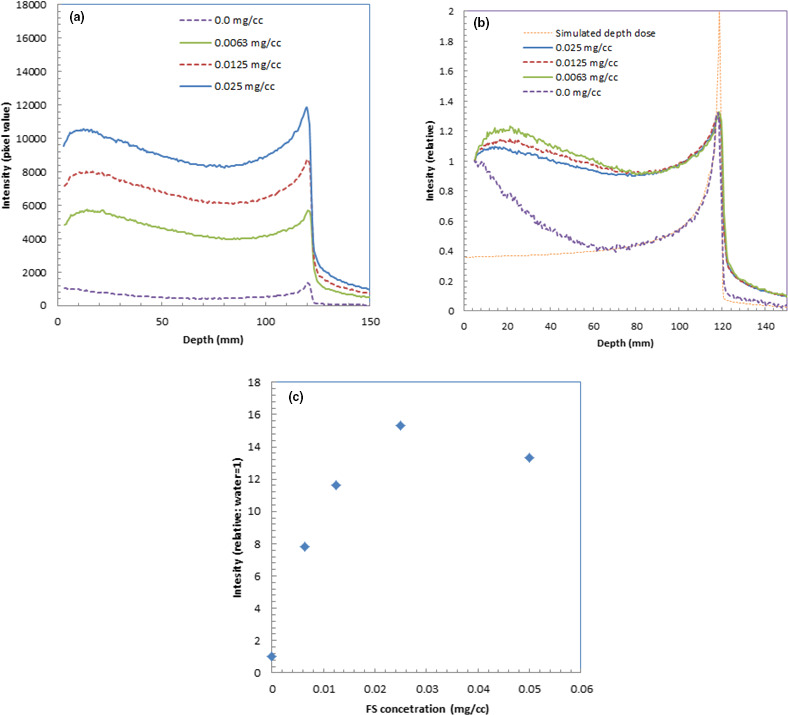
Depth profiles of the measured luminescence images of water during irradiation of 241.5‐MeV/u carbon ions with fluorescein at different concentrations (a), relative depth profiles of the measured luminescence images of water with fluorescein at different concentrations (b), and relative intensity of the depth profiles as a function of fluorescein concentration (c).

We show the depth profiles of the measured luminescence images of water with fluorescein at different concentrations in Fig. 6(a). We could observe the luminescence intensity increase as the fluorescein concentration increased.

Figure 6(b) shows the relative depth profiles of the measured luminescence images of water normalized at the shallow part of the depths with a calculated depth dose profile. These relative depth profiles were normalized at the entrance of the phantoms for the measured images. The calculated dose was normalized at the middle part (~70 mm depth) of the luminescence image of water. The depth profiles had similar shapes except for water without fluorescein (concentration 0.0 mg/cm^3^). The depth profile for water showed a thinner Bragg peak and high intensity in the shallow area due to the Cherenkov light from the secondary electrons. The depth profiles with fluorescein showed higher intensities around the mid‐point of the depths (~20–120 mm).

Figure 6(c) shows the relative intensity of the measured luminescence images of water as a function of fluorescein concentration, where the intensity of water is normalized to 1.0. These intensities were evaluated by averaging at depths from 0 to 170 mm. The luminescence intensity was more than 10 times higher than water for the images with fluorescein concentrations greater than 0.0125 mg/cm^3^.

Table 3 lists the ranges estimated from the luminescence images of water and fluorescein at different concentrations. Ranges were estimated at 80% of the peak for the profiles. The ranges were matched with the measured luminescence images of water, fluorescein at different concentrations, and simulation within an error of 1 mm.

**Table 3:** Ranges estimated from luminescence images of water and fluorescein (FS) at different concentrations and simulation for carbon ions.

	Water	FS: 0.0063 mg/cm^3^	FS: 0.0125 mg/cm^3^	FS: 0.025 mg/cm^3^	Simulation
Range	119 mm	120 mm	120 mm	120 mm	119 mm

**Fig. 7:**
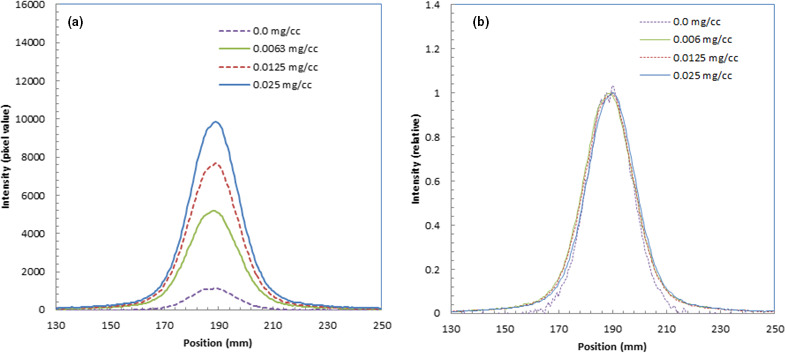
Lateral profiles of measured luminescence images of water during irradiation of 241.5‐MeV/u carbon ions in Bragg peak area with fluorescein at different concentrations (a) and relative lateral profiles of the measured luminescence images of water normalized at the peaks (b).

Figure 7(a) shows the lateral profiles of the measured luminescence images of water during irradiation of 241.5‐MeV/u carbon ions in the Bragg peak area (118‐mm depth) with fluorescein at different concentrations. We could observe the luminescence intensity increase as the fluorescein concentration increased.

We show the relative lateral profiles of the measured luminescence images of water normalized at the peaks in Fig. 7(b). The widths were the same as those with fluorescein at different concentrations, and these widths were slightly wider than that of water.

Table 4 lists the beam widths in FWHM estimated from luminescence images of water and fluorescein at different concentrations for carbon ions. The widths with fluorescein were 2–3 mm wider than that of water.

**Table 4:** Beam widths estimated from luminescence images of water and fluorescein (FS) at different concentrations for carbon ions.

	Water	FS: 0.0063 mg/cm^3^	FS: 0.0125 mg/cm^3^	FS: 0.025 mg/cm^3^
FWHM	19 mm	22 mm	21 mm	21 mm

### 3.C Light spectra measurement of fluorescein water by irradiation of UV light

**Fig. 8:**
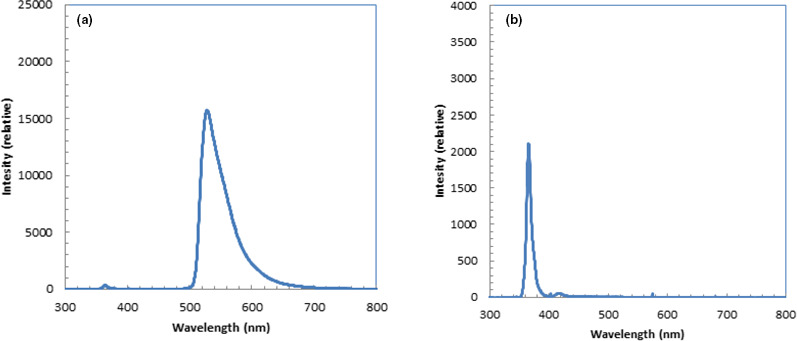
Light spectra of fluorescein water (a) and water (b) by irradiation of UV light with wavelength of 365 nm.

The measured light spectrum for fluorescein water is shown in Fig. 8(a). We could observe that the light distribution from 500 to 700 nm peaked at 530 nm. We could also observe a small peak at 365 nm from the irradiated UV light.

The measured light spectrum for water is shown in Fig. 8(b). We could observe a peak only at 365 nm from the irradiated UV light.

## 4 DISCUSSION

In this study, we confirmed an increase in luminescence intensity by adding fluorescein to water for both protons and carbon ions. The intensities of luminescence images increased to more than 10 times larger than that of water for fluorescein concentrations greater than 0.0125 mg/cm^3^. Therefore, fluorescein can be used for the luminescence imaging of water in a shorter time.

For the luminescence images of protons, the range for the measured luminescence image of water matched those of fluorescein at different concentrations (Table 1). These ranges also matched the simulated range. Thus the luminescence imaging of protons with fluorescein can at least be used for range determination at shorter imaging times.

The depth profile for water derived from the luminescence image of protons in Fig. 3(b) shows a slightly lower Bragg peak and slightly higher intensity around the mid‐point of the depth compared with those derived from the images with fluorescein. This was probably because the depth profile for water derived from the luminescence image contained higher Cherenkov light from the prompt gamma photons than did that for the luminescence of fluorescein water. With proper correction of Cherenkov light from the prompt gamma photons, these differences were eliminated.^3^ The luminescence images with fluorescein water contained some Cherenkov light from the prompt gamma photons, but the contamination from this light was small, making the correction for Cherenkov light easier than that for water. Because the wavelength of fluorescein water is from 500 to 700 nm as shown in Fig. 8(a), using a bandpass or long‐pass filter would block the Cherenkov light at shorter wavelengths and possibly reduce the Cherenkov‐light components from the prompt gamma photons.

For the luminescence images of water for carbon ions, we could observe that the luminescence intensity increased by adding fluorescein to water as shown in Fig. 6(a). From the luminescence images of carbon ions, the ranges could be estimated from the measured luminescence images of fluorescein, although the ranges were 1 mm longer than that of the simulated results listed in Table 3.

The depth profile shapes with fluorescein for carbon ions were significantly different from that of water; the depth profiles with fluorescein showed higher intensities around the mid‐point of the depths (~20–120 mm) as shown in Fig. 6(b). This was probably because the angular dependency of Cherenkov light was reduced or nearly eliminated by adding the fluorescein to water, and the Cherenkov light directed forward was thus detected by the CCD camera. The Cherenkov light from the lighter ion particles produced by nuclear spallation^17^ may have contributed to the increase in intensity with fluorescein. Since estimation of the depth profile shapes with fluorescein was complicated, it may be difficult to obtain depth dose profiles from them by correction using simulated data, which was successful for the correction of the luminescence images of water for carbon ions.^4^ Taking measurements at a lower energy than the Cherenkov‐light threshold may explain the distorted depth dose profiles of carbon ions shown in Fig. 6(a).

There are some differences in relative intensity of the depth profiles, as a function of fluorescein concentration, between protons and carbon ions as shown Figs. 3(c) and 6(c). The differences were probably due to the effect of contamination by Cherenkov light at shallow depths and the difference in precipitation between these two conditions. Furthermore, the luminescence‐enhancement effect by the fluorescein water does not linearly change with the concentration. This was probably due to saturation of the fluorescein water's solubility. At a fluorescein concentration higher than 0.025 mg/cm^3^, significant precipitation of fluorescein was observed in the water phantom. The proper concentration of fluorescein water would be ~0.01 mg/cm^3^, which could increase luminescence by ~10 times without any precipitation of fluorescein in the water phantom.

## 5 CONCLUSION

We confirmed an increase in the intensity of the luminescence of water by adding fluorescein for protons and carbon ions. With a small amount of Cherenkov‐light contamination in the images, such as that by protons, the depth profiles derived from the luminescence images with fluorescein were similar to that of water and will be used for range or dose determination. With the contamination of Cherenkov light such as that by carbon ions, the depth distributions were different from that measured with water, while ranges derived from the images were slightly larger.

## CONFLICT OF INTEREST

No conflicts to disclose with the authors.

## Data Availability

Data are available on request from the authors.
